# Supplementary material for: Are carbon emissions trading and green financial instruments synergistic? -Comprehensive quantitative research based on content analysis
Source: PLoS One. 2024 Mar 7;19(3):e0298601. doi: 10.1371/journal.pone.0298601 (PMC10919601; doi:10.1371/journal.pone.0298601)
Supplement: S1 Data — (ZIP) [file pone.0298601.s001.zip › Data/地级市Z_F工作报告.zip!/║╙▒▒/║Γ╦«╩╨/└┤╫╘╣1⁄2╓┌║┼í╛┬φ┐╦╩2╛▌═°í┐_2015.docx]

各位代表：

现在，我代表市人民政府向大会作工作报告，请予审议，并请市政协委员和列席会议的同志提出意见。

一、2014年工作回顾

过去一年，是我市面临形势复杂严峻、困难挑战十分突出的一年，也是跨越赶超、改革发展迈出重要步伐的一年。在省委、省政府和中共衡水市委的正确领导下，全市人民认真贯彻党的十八大和十八届三中、四中全会精神，以习近平总书记系列重要讲话为指引，紧紧围绕“强势开局、跨越赶超，全面建成小康社会”总要求，以开展党的群众路线教育实践活动为动力，以综合配套改革为统领，全力以赴抓改革、打基础、造环境、铸优势、求赶超，努力实现市五届人大二次会议确定的任务目标。

—-经济发展逆势上扬。全市经济在逆势中上扬，在困境中突破，继续保持增速领先、总量晋位的良好态势。全年完成生产总值1139亿元，较上年增长8.2%。固定资产投资945.2亿元，增长20.1%。全部财政收入148.6亿元，其中公共财政预算收入79.7亿元，分别增长15.1%和16.3%。规模以上工业增加值447亿元，增长9%。社会消费品零售总额546.9亿元，增长12.6%。各项存款余额超2千亿元，各项贷款余额超千亿元，存贷比突破50%。统计的17项主要经济指标中，有5项指标增速位居全省第一，有8项指标增速位居全省第二。规模以上工业增加值、新增贷款实现总量晋位。固定资产投资、全部财政收入和公共财政预算收入等基本实现三年翻番。桃城区、工业新区全部财政收入超30亿元。继安平、景县之后，枣强、故城、冀州全部财政收入突破10亿元。

—-质量效益稳步提高。产业结构发生积极变化，第三产业增加值占生产总值比重比上年提高3.5个百分点。税收增长高于全部财政收入增长，税收占全部财政收入增收的比重达到92.3%。城镇化率预计达到43.9%。省市重点项目中战略性新兴产业、装备制造业、服务业项目占比达77.4%，提高17.4个百分点。

—-人民生活持续改善。城镇新增就业4.2万人。城乡居民人均可支配收入分别达到19614元和8104元，增长10.1%和12.8%。居民消费价格指数上涨2.1%。

一年来，我们主要做了以下几个方面的工作。

(一)沉着应对经济下行压力，力促经济平稳较快发展。去年我市经济指标增速不同程度趋缓回落，面对严峻形势，我们主动适应新常态，及时采取一系列有针对性的政策措施，抓投资、扶企业、兴产业。

强力推进投资和项目建设。牢牢抓住投资这个稳增长的关键，拓宽项目建设领域，完善项目谋划和推进机制，固定资产投资逐月回升，省市重点项目完成投资421.7亿元，超年计划5.3个百分点。筛选确定了总投资176亿元的两批共33项政府推进重点工程，完成投资57亿元。邢衡高速衡水段一期、大广高速衡水南连接线、石黄高速武强连接线、衡德公路衡水北口至周通段(东北环)等建成通车，实现了市区4个进出口全部为一级以上公路，为形成市区高速环线奠定了基础。衡井线主体工程完工，石济客专、衡德高速故城支线、邢衡高速二期等开工建设，建设农村公路434.8公里 天火大道辰东。海伟聚丙烯和阳煤乙二醇两项超百亿元项目顺利推进，泰华伟业服务机器人、亚森工业机器人、澳泰环保节能设备等一批高科技项目开工建设，养元植物蛋白饮料、奥冠高性能蓄电池等106个项目建成投产。

加大扶持服务企业力度。牢牢抓住实体经济这个稳增长的基础，开展走百企访百家系列调研活动，现场协调解决企业生产经营中的突出困难和问题。落实中小企业发展扶持政策，出台进一步减轻企业负担等20项措施，依法依规减免税总额超过5亿元。加强要素保障。争取省下达我市新增用地指标10781亩，为49个重点项目安排土地3800亩。13个项目被列为省重点在建和前期项目，争取省用地指标1323亩。全年新增贷款185.5亿元，争取上级各类专项资金116亿元。新增阜城尚真新材料、饶阳众悦等上市企业10家，超过往年总和。民营经济增加值达到797.4亿元，增长8.5%。

(二)着力转方式调结构，推动经济提质增效升级。坚持把提高质量效益放在核心位置，在加快赶超的同时更好推动发展转型，努力打造衡水经济升级版。

现代农业强市实现新突破。以提升农业综合生产能力、确保粮食安全和主要农产品有效供给为目标，以土地流转为抓手，坚定不移发展规模农业、法人农业、科技农业。粮食总产72.8亿斤。土地流转面积299.8万亩，占家庭承包总面积的38.2%，提高11.1个百分点。新增3000亩以上规模经营典型40个，新增注册家庭农场2651家，新登记农民合作社3220家。144个农业产业化重点项目完成投资180.5亿元。武强北大洼、故城东大洼、景县津龙、枣强唐林、安平京安等现代农业示范区建设加快推进。饶阳县、故城建国镇等智慧农业试点成效显现。阜城县被农业部命名为全国有机瓜菜种植基地示范县。深州蜜桃、大营裘皮、饶阳甜瓜、饶阳葡萄地理标志产品保护获国家质检总局批准，填补了我市地理标志产品保护的空白。主要农作物耕种收综合机械化水平达到85%，全国农机深松整地作业现场会在我市召开。

工业转型升级迈出新步伐。开展战略导向产业选择研究，实施“1+10”行动计划，努力培育立市主导产业和行业巨人。新增规模以上工业企业153家。新创中国驰名商标1件、省著名商标55件，新创省名牌产品46项、优质产品24项。启动全国工程橡胶产业知名品牌创建示范区筹建工作。养元公司销售收入突破百亿元，上缴税金13.2亿元。“有中生新”改造提升传统产业，大力推进企业技术改造和管理改造，完成工业技改投资461.1亿元，增长27%，居全省第二。“无中生有”培育壮大新兴产业，高新技术产业投资增长63%。全市高新技术企业达到73家，新增11家；科技型中小企业达到555家。成立了科技特派员创新创业协会。12家企业被评为省级百家两化融合重点企业，丰泽橡胶公司被认定为国家火炬计划重点高新技术企业。5个国别园(地区园)列入省重点支持园区，景州高新技术产业开发区被批准为省级高新技术产业开发区，大营经济开发区被批准为省级经济开发区。

现代服务业发展取得新进展。出台实施加快服务业发展的指导意见，服务业实现增加值395.2亿元，增长11.2%。安平内陆港、衡德综合物流园等5个物流园区加快建设。电子商务交易额突破400亿元，安平玛世电子商务公司被列为国家级电子商务示范企业，大营裘皮电子商务基地被授予首批全省电子商务示范基地。旅游业发展加快，全年接待游客838万人次，旅游业总收入53.1亿元，分别增长19.7%和25.8%。周窝音乐小镇获评“牵手2014中国最美村镇”，饶阳民族乐器产业园被评为省十大文化产业聚集区，闾里?婚冠里入选省十大文化产业项目。金融业发展增速高出经济发展增速7.7个百分点，实现税收12.5亿元。邢台银行、首创证券、广发证券、永安信基金落户我市。阜城农商银行、衡水农商银行先后挂牌。安平惠民村镇银行、深州丰源村镇银行相继开业。我市被省政府授予金融生态市称号。

(三)积极推进新型城镇化建设，加快城乡一体化发展。坚持扩容与提质并重，优化城乡布局，提高建管水平。

中心城市功能日趋完善。实施市污水处理厂升级改造、前进街明渠污水治理、滏阳河便民服务设施建设、街道便道硬化、垃圾池改造、街头游园提升、小街小巷改造等一批城市功能完善工程。硬化、绿化市区主干道路裸露地块40万平方米，市区榕花大街改造提升道路绿化工程开工建设。新增集中供热284万平方米，新建换热站18座。新建垃圾压缩站30座，主城区开放式地下垃圾池全部取消，市医疗废物处置中心开工建设。扎实推进河东旧城区3个片区改造。滨湖新城建设有序推进，衡水第一中学建成投用。加强数字化城管平台建设，省市数管中心实现信息对接。

县城综合承载能力明显提高。开展县城建设三年攻坚战，各县市确定实施项目111个，完成投资79亿元，建成综合性公园22个，新增乔木35.5万株、绿地271公顷。继冀州、深州、武邑之后，阜城、枣强、安平通过省级园林县城验收。阜城、枣强荣获省政府人居环境进步奖。安平成功创建省级卫生城。

农村环境面貌显著提升。积极推进工业园区、农业园区和新型农村社区“三区同建”，建设5万平方米以上联建社区46个，涌现出枣强李进伯社区、故城小马坊中心村等一批先进典型。投资5.4亿元，完成280个重点村提升改造。

(四)实施生态建设攻坚突破，不断创新发展优势。把生态建设放在关系发展全局的战略高度，进一步塑造、培育和拓展我市的生态优势。

坚决打好大气污染防治攻坚战。狠抓压煤、治企、降尘、控车、增绿等重点工作，综合整治大气污染。淘汰燃煤锅炉274台，压减燃煤20万吨，超额完成省定任务。关停排污企业245家，停产整顿121家，限期治理520家，淘汰黄标车13145辆。集中开展秸秆禁烧、散烧燃煤管控、扬尘治理等攻坚战，圆满完成APEC会议期间空气质量保障任务。细颗粒物(PM2.5)、可吸入颗粒物(PM10)、二氧化硫、二氧化氮、一氧化碳平均浓度分别下降11.5%、11.5%、38.2%、6.5%、21%，城区环境空气质量综合指数9.85，下降16%。预计全市单位生产总值能耗下降4.5%以上，超额完成年初目标任务。

扎实推进地下水超采综合治理试点工作。作为全市的“一号工程”，争取上级投资37.68亿元，579个标段全线开工，完成投资26.5亿元，半数土地和农户受益。全省地下水超采综合治理现场会在我市召开。投资46.2亿元的南水北调中线配套工程全面开工。人工影响天气作业成效明显，增加有效降水5100万立方米。衡水湖国家级自然保护区范围和功能区划调整获国务院批准，衡水湖获得国家良好湖泊生态修复项目支持。

大力开展造林绿化。坚持产业化、规模化、市场化造林，全年完成造林33.3万亩，提前一年实现“一人一亩林”目标。衡水湖周边建成3000亩以上林业园区11个，其中冀州小寨森林公园植树1.6万亩。全省公路绿色廊道建设现场会在我市召开。

(五)全面深化改革开放，激发发展活力动力。坚持改革破解难题、开放引领发展，以改革先行一步推动发展快赶几步。

大力推进综合配套改革。认真落实省委、省政府《关于衡水市开展综合配套改革的意见》，积极稳妥推进重点领域改革。市本级取消下放行政审批事项621项，取消下放比例达75.8%。积极推进“三级平台”、“两个代办”，市县政务服务中心得到提升，乡村便民服务中心实现全覆盖。农村土地承包经营权确权登记110.2万亩，占承包耕地总面积的13%，居全省首位。深化注册资本登记制度改革，推行“先照后证”改革和企业年度报告公示制度，在全省率先实行工商营业执照、组织机构代码证和税务登记证“三证合一”。积极推动全民创新创业，建立衡水职业技术学院师生创业孵化基地、衡水学院工业新区产学研基地。新增市场主体3.59万户，其中企业1.16万户，全市市场主体总量达16.16万户。

积极融入京津冀协同发展。明确了“一枢纽四基地”功能定位，大力实施“三个一百”工程。与北京、天津洽谈引进项目94项，总投资996亿元。“蓝火计划”衡水基地挂牌成立。成功举办百家纺织服装企业进衡水推介活动，全省首家承接全国纺织产业转移试点园区正式落户工业新区，北京威克多等一批企业落地建设。成功举办衡水湖国际商务洽谈会等招商活动，签约项目163项，引进资金1151.7亿元。衡水海关开关运行。

(六)切实保障改善民生，保持社会和谐稳定。财政用于民生的支出达到190.6亿元，增长35.3%，占公共财政预算支出的81.3%。市委、市政府承诺的13件民生实事基本完成。

抓好就业和社会保障工作。实施大学生创业帮扶工程，开展“一条龙”创业服务和创业培训。开工建设保障性住房22375套，竣工10019套，完成省定目标。城镇居民医疗保险年人均财政补助提高到320元。新农合筹资标准提高到390元，新农合大病保险工作全面推开。85岁以上长寿老人高龄补贴实现全覆盖。向7671名贫困重度残疾人发放生活补贴。试点推进“救急难”工作，为特困家庭应对特殊困难提供帮助。

提升公共服务质量水平。加强公共文化设施建设，现代公共文化服务体系更加完善。衡水学院西校区、冀州第一中学、前进街小学、东方太阳城小学建成投用，新苑小学、胜利小学和第十中学改扩建工程主体完工或部分投入使用。衡水中学、衡水二中等中学办学水平进一步提升。2849个农村标准化卫生室全部建成。衡水湖国际马拉松赛暨全国马拉松锦标赛(第5站)被评为金牌赛事和2014中国体育旅游精品赛事。“篮球城”创建扎实推进，市区新建篮球场70余块。整合各类扶贫和涉农资金38亿元，10万贫困人口实现稳定脱贫。投资1.8亿元，解决了39.4万农村人口饮水安全问题。

加强和创新社会管理。完善信访和调解联动工作体系，做好涉军维权工作，有效化解矛盾纠纷，促进了社会和谐稳定。深入开展食品药品安全专项整治和餐桌保卫战，食品药品监管体系进一步完善。加强安全生产监管，严厉打击非法生产行为，连续两年未发生较大以上生产安全事故。深入推进平安衡水建设，保持严打整治高压态势，全市社会治安大局持续稳定。饶阳“三角地带”打击治理行动入选2014年度省十大法治事件。主城区和各县市城镇实现常态化武装巡逻，人民群众安全感不断提升。开展了深州“南街村”综合治理，为依法解决农村组织涣散、秩序混乱、非法占地等问题积累了经验。

按照中央和省、市委统一部署，扎实开展党的群众路线教育实践活动，广泛听取各方面意见和建议，认真对照检查，制定整改措施，狠抓整改落实，解决了一批群众反映强烈的突出问题。严格执行“八项规定”和“约法三章”，全面清理和处置违规公务用车、超标办公用房、“吃空饷”人员。市直机关“三公”经费支出较上年压减19.6%。市政府印发文件减少11.3%。密切联系群众，改进工作作风，961890和12345两条群众服务热线共接听受理来电、信件76700多件(次)，办结率99.8%。探索实行了土地管理委员会委员实名提出宗地出让底价集体决策办法，机关标准化管理工作扎实推进，制定了《重大项目风险评估制度》、《清理闲置土地制度》、《行政机关限时办结制度》、《关于加大行政问责力度提高政府部门执行力的意见》等，提升了政府管理的科学化、标准化、法治化水平。加强稳增长、调结构、促改革、惠民生政策执行和财政预算执行情况的审计监督和督查落实。自觉接受人大法律监督、工作监督和政协民主监督，办理人大代表建议和政协提案439件，办结率100%。

国防动员和双拥共建深入开展，国防后备力量建设得到有力加强，军政军民团结局面进一步巩固。人口计生、民族宗教、新闻广电出版、外事侨务、涉台事务、人民防空、气象地震、地理信息、邮政通信、援疆援藏、妇女儿童、老龄、残疾人、统计、史志、档案等工作都取得新成绩。

成绩来之不易。这是省委、省政府和市委正确领导的结果，是全市人民同心同德、克难奋进的结果，是人大代表和政协委员监督、支持的结果，是社会各界和衷共济、通力协作的结果。在此，我代表市政府，向在全市各条战线辛勤工作的建设者、劳动者，向人大代表、政协委员，向离退休老同志，向各民主党派、工商联、无党派人士和人民团体，向驻衡部队、武警官兵、政法干警和中省直驻衡各单位，向积极参与、关心支持衡水发展的各界朋友，致以崇高的敬意和衷心的感谢!

各位代表，我们也清醒地认识到，我市发展中仍然存在不少困难和问题，政府工作与人民群众期待还有差距。去年地区生产总值、外贸进出口总额没有达到预期目标。我市经济总量不大、产业层次不高、创新能力不足等问题尚未根本解决。宏观经济下行压力传导加大、部分企业生产经营困难、财政收支矛盾突出等问题日益凸显。大气等环境污染依然严重。政府职能和工作作风转变还不到位，一些部门和工作人员依法行政和服务意识淡薄，消极腐败现象还不同程度存在。对此，我们一定高度重视，勇于担当，采取有力措施，认真加以解决。二、2015年工作总体要求

今年是全面深化改革的关键之年，是全面推进法治衡水建设的开局之年，也是全面完成“十二五”规划的收官之年。当前，国内外发展形势依然错综复杂，经济下行压力依然较大，全省正处于转型升级、爬坡过坎的关键时期，我市稳增长调结构任务艰巨。同时也要看到，支撑我市经济较快增长的基础比较扎实，有很多现实的、可争取的、能把握的重大机遇。京津冀协同发展战略全面实施，一批重大事项、重点项目加快推进，我市在区域发展中的潜在优势正在逐步显现。综合配套改革全面深化，新型城镇化加快推进，越来越多的改革红利、城镇化红利将加速释放，发展的基础条件和整体环境将进一步改善。这几年新上的一批项目逐步见效，企业经营管理水平不断提升，有利于增强抗风险能力，形成新的增长点。特别是通过开展党的群众路线教育实践活动，干事创业氛围日益浓厚，为我市跨越赶超、绿色崛起提供了重要保障。

政府工作的总体要求是：全面贯彻党的十八大和十八届三中、四中全会以及习近平总书记系列重要讲话精神，认真落实中央和省委、省政府以及市委重大部署，以“强势开局、跨越赶超，全面建成小康社会”为总要求，坚持稳中求进工作总基调，坚持以提高经济发展质量效益为中心，全面深化改革，突出创新驱动，注重改善民生，推进依法治市，实现新常态下经济平稳较快发展和社会和谐稳定。

全年经济社会发展的主要预期目标是：生产总值增长8%；固定资产投资增长18%；全部财政收入和公共财政预算收入分别增长10%；实际利用外资增长5%；外贸进出口总额和出口总额分别增长6%；社会消费品零售总额增长11%左右；城乡居民人均可支配收入分别增长9.5%和10.5%；细颗粒物(PM2.5)浓度下降4%；居民消费价格指数涨幅控制在3%左右；城镇登记失业率控制在4.5%以内；人口自然增长率控制在8.1‰以内；单位生产总值能耗和主要污染物排放完成省定目标 昆仑小说。上述指标的提出，体现了“强势开局、跨越赶超”要求，与“十二五”规划目标、全面建成小康社会目标、综合配套改革目标相衔接，符合我市实际和社会预期，可以满足扩大就业和居民收入增长的需要，有利于为调整经济结构和深化改革开放腾出空间。

做好今年的各项工作，必须准确把握经济发展新常态的特征和要求，做到观念上适应、认识上到位、方法上对路、工作上得力，牢牢掌握经济发展主动权。要坚持跨越发展，主动作为。充分利用我市产业结构轻、动力转换快等有利条件，增效扩量，效益能搞多好就搞多好，速度能搞多快就搞多快，缩小总量差距，倒逼质量提升，努力实现“弯道超车”。要坚持转型发展，保持定力。牢固树立转型才能更好发展、后发也要高点起步的理念，以“功成不必在我”的历史责任感，坚定不移转方式调结构，扎扎实实打基础增后劲，走出发展新路，抢抓发展先机。要坚持创新发展，激发动力。把创新驱动作为贯穿当前和今后一个时期全市经济社会发展的一条主线，优化提升存量，培育发展增量，打造新产业、新业态、新商业模式，积极培育新的经济增长点，促进经济增长从要素驱动为主向创新驱动为主转变，推动产业结构加快由中低端向中高端迈进。

三、全力以赴稳增长调结构

(一)充分发挥投资的关键作用。坚持增投入、上项目，以项目引领带动经济发展扩量提质。重大基础设施项目。重点抓好石济客专、邢衡高速二期、衡德高速故城支线等在建项目，富德公路、大广高速衡水湖连接线、106国道改线开工建设。加大地下水超采综合治理和南水北调中线配套工程建设力度，确保如期完成。谋划推进衡水机场、冀州通用机场等项目。落实县乡主体责任，做好农村公路改造养护工作，提升道路通行能力，全年改造农村公路500公里。实体经济项目。投资增速保持在22%以上，力争10亿元以上重点项目达到110个。实施“四个一百”工程，全市重点抓好市级以上计划开工项目100项，续建保投产项目100项，推进前期项目100项，谋划立市立县重大项目100项。力促投资22.3亿元的烨和祥生物玉米纤维、投资20.8亿元的华尔特高科技丝网产业综合体、投资20.8亿元的国机集团清洁能源及冷能装备等项目早日开工，推进投资30亿元的安德森物联网智能家居、投资22亿元的衡美高档纱线、投资11.5亿元的冀中能源大有公司矿用智能装备等项目前期工作。加大招商引资力度。浓厚招商氛围，创新招商方式，打造优良环境。继续瞄准国内外500强企业开展定向招商、委托招商和驻点招商。充分利用5?18经洽会等平台，办好衡水湖国际商务洽谈会、安平国际丝网博览会等经贸洽谈活动，加强与京津、长三角、珠三角等重点地区的合作。切实做好项目要素保障。发挥土地政策导向功能，用好增减挂钩政策，促进土地节约集约利用。对工作实、效益好、推进快的直接支持、重点保障。改革投融资体制，鼓励民间资本发起设立创业投资基金和股权投资基金，推广政府与社会资本合作(PPP)模式，吸引更多社会资本投入城市基础设施建设和运营。

(二)推进工业转型升级。深入开展工业转型升级年活动，力争规模以上工业增加值增长11%，工业投资增长20%以上。加快培育立市主导产业。围绕“6+3”主导产业发展规划，继续推进“1+10”行动计划，集中生产要素精准支持，促进主导产业、重点企业做大做强。继续抓好企业技术改造和管理改造。开展规模以上工业企业“1+1”技改活动，组织实施企业经营者素质提升、现代企业制度建设和企业整合重组三大工程，帮助和引导企业完善公司法人治理结构，提升经营者整体素质。加大铁塔钢构产业整合力度，谋划建设国家铁塔钢构综合产业示范园区。深入实施科技创新行动计划。确保机器人项目投产达效，带动智能制造产业形成规模。以食品及生物制品制造业和纺织服装制造业为试点，探索主导产业培育发展的思路目标、路径方法和政策措施，建设一批产业公共服务平台。抓好橡胶、玻璃钢、橡塑、铸造等产业集群两化融合公共服务平台建设，促进传统特色产业转型升级。积极推进科技成果转化收益分配改革，促进技术资本化、人才股份化，支持收益分配向发明者倾斜，加快转化科技成果。加大企业帮扶力度。完善订单式服务制度，把走百企访百家活动制度化。支持各类经济组织联合重组，培育和发展大型企业集团。加大“个转企、小转一、下转上”工作力度，确保全年新增规模以上工业企业150家以上。深入开展大众创业万众创新活动。扎实开展扶助小微企业专项行动，大力推行“三证合一”，实行初次创业无约束、无障碍、保姆式服务机制，最大程度激发创业创新热情，力争新增市场主体2万个以上。加强政府债务管理，实施企业信用培育双百工程，防范和化解债务金融风险。抓好工业园区建设。开展园区活力增强年活动，提高园区综合服务能力和水平。深化园区体制机制改革，先期开展聘任制及薪酬制度改革。工业新区谋划实施西区改造，争取晋升国家级开发区。提高开放型经济发展水平。加大先进技术和设备引进力度。加强丝网、化工、皮毛等外贸转型升级基地建设。抓住“一带一路”和省政府推动加强与中东欧国家全面合作重大机遇，鼓励有条件的企业“走出去”，扩大对外投资和贸易。

(三)加快建设现代农业强市。坚定不移走中国特色新型农业现代化道路，加快转变农业发展方式，力争现代农业强市建设迈出更大步伐。稳定粮食生产。重点抓好节水品种和节水技术推广、高产创建、整建制推进吨粮田建设等工作，全市粮食播种面积保持在800万亩左右，总产稳定在70亿斤以上。推动土地经营权规范有序流转。加快推进农村土地承包经营权确权登记颁证试点工作，全市确权面积达到承包耕地总面积的60%以上。积极培育专业大户、家庭农场、农民合作社等新型市场经营主体，力争培育市级示范性家庭农场100家以上，新增家庭农场1000家，年内农村土地流转比例达到40%。加强市县乡三级土地流转服务平台建设，提升土地流转管理服务水平。大力推动土地合作社统一对外招商，开展规模经营，为各类资本投资现代农业铺平道路。建立县级农村产权交易市场，开展城乡统一的土地市场改革试点，创新未利用地综合开发机制。推动流转面积3000亩以上的规模经营主体率先建成现代农业园区。提高农业产业化水平。以优质奶业基地和标准化规模养殖场建设为重点，进一步增强畜禽综合生产能力。以设施蔬菜为重点，以标准化蔬菜基地创建为抓手，努力构建现代蔬菜产业体系。打造一批销售收入超10亿元的产业化龙头企业，农业产业化经营率达到68%以上。积极开展土地经营权向金融机构抵押融资改革试点。推进供销社综合改革，构建新型农业社会化服务体系。

(四)加快服务业发展。像抓工业一样抓服务业，坚持生产性服务业与生活性服务业并重，着力推动服务业扩规模、优结构、提质量。大力促进物流业、电子商务和金融业发展。围绕打造京津冀区域交通物流枢纽定位，重点抓好衡德综合物流园、衡东物流园、新发地农副产品物流园、安平内陆港等物流园区建设。依托丝网、医药、皮毛、建材等打造特色产业物流基地。结合农产品基地建设，在饶阳、阜城发展一批果蔬物流企业和园区。继续抓好安平丝网、景县胶管、枣强裘皮、冀州暖气片、饶阳蔬菜、阜城特色农产品等6个特色电子商务平台建设，争列省农村电子商务示范县。推进金融业健康稳定发展，深化金融改革创新，建立健全地方金融监督管理体制，争取引进5家以上域外金融机构，积极推进枣强、武邑、饶阳、安平、故城、景县6家县农联社股份制改造工作。加快企业上市步伐，力争新上市企业10家以上。加快推进旅游文化健康产业融合发展。启动全市旅游业发展总体规划编制工作，加大旅游项目建设力度，完善旅游基础设施和公共服务设施，改善旅游环境，旅游业总收入增长18%以上。按照国家5A级旅游景区标准建设衡水湖景区，推进衡水湖环湖健步路和自行车道等基础设施建设，打造全国知名的健步休闲养生旅游目的地。以推动环衡水湖文化产业聚集区和加强董子文化旅游开发为重点，加快汉民俗文化小镇、周窝音乐小镇、董子故里、冀州古城恢复等重点文化旅游项目建设。深入挖掘冀派内画、侯店毛笔、宫廷金鱼、武强年画、陈集剪纸等传统文化资源，积极开发特色旅游商品。发展老白干、六个核桃、深州蜜桃观光园、饶阳现代农业产业园等工农业旅游。改造和提升传统服务业。推进一批城市商贸综合体和一县一特色街区建设。推动各类传统市场、商业企业创新经营模式，促进传统商贸流通方式向现代商贸流通方式转变。支持商铺发展夜经济。加强限上企业入统工作，确保完成三年新增限额以上住餐批零企业500家任务。

四、稳步有序推进新型城镇化

把新型城镇化作为新常态下经济发展重要的引爆点和支撑点，加快中心城市和县城扩容提质步伐，打造生态湖城、美丽衡水。

(一)构建城乡一体的城镇化布局。树立“大衡水”理念，促进中心城市和县城、乡镇以及农村社区合理分工、协同发展。实施中心城市带动战略，以“一湖三区”为主体组团式发展中心城市，继续大力推进滨湖新城棚户区改造项目、南部新区建设和河东旧城区改造。实施大县城战略，全面提升县城建设标准，加快县城扩容升级步伐，突出文化元素融入，打造宜居宜业特色县城。加快向重点镇下放经济社会管理权限步伐，有条件的建制镇设立一级财政，建设产业小镇、明星小镇、特色小镇，增强乡镇承载能力和发展活力，突出打造30个重点镇。做好大营镇和大营经济开发区、龙华镇和龙华台湾工业园“镇区合一”试点工作。以“三区同建”为抓手，加快发展农村新型社区，每个县市区至少培育5个“三区同建”示范点。以衡水湖片区36个村为重点，打好生态牌，打造湖光秀色、诗意乡村。全市再确定140个左右的重点村，争取每县都有村庄进入全省美丽乡村行列。

(二)加快城镇公共设施建设。加大城镇市政基础设施建设力度，抓好榕花大街改造提升、滏阳河市区段综合提升、南部新区基础设施配套、市民中心、体育中心、哈院新院区等一批重点项目，积极推动火车站改造、高铁新站建设、桃冀城际轨道交通等项目前期工作。坚持旧城改造与新区建设互动，中心城区拆迁改造达到30%，完成60%旧小区改造。规范完善房地产领域相关政策，支持房地产业平稳健康发展 侯卫东官场笔记。创造条件，争取每个镇区都有房地产项目。争取衡水垃圾发电厂改造投用，市区、县城生活垃圾无害化处理率分别达到95%和90%以上、污水处理率分别达到90%和80%。

(三)提高城市管理水平。树立和强化城市理念，加大财政投入，全面提升城市规划建设和精细化管理水平。完善规划体系，抓好冀州、景县“四规合一”试点。推动以数字化社区为重点的智慧城市建设，探索在安全保障、教育管理、社区服务等民生领域扩大信息消费的有效途径，让群众更好享受信息化发展带来的好处。抓好冀州、安平智慧城市试点。完善数字化城市管理平台，每个县市都要建成数字化城管平台。推行地下综合管廊建设模式，对埋入地下的各类管线和设施实行统一管理和维护。开展治脏治乱专项行动，加大马路市场、停车场等整治提升力度，加强小区物业管理，开工建设建筑垃圾处置场，城市道路机械化清扫率达到50%以上。加快园林城市创建步伐，主城区要深入持久地开展容貌整治、绿化美化、品位提升等工作，积极申报国家园林城市；所有县城今年都要建成省级园林城市。

(四)加速城镇人口聚集。扎实推进户籍制度改革，建立城乡统一的户口登记制度，完善居住证制度和实际居住人口登记制度，放开城市和建制镇落户限制，引导农村人口自愿、有序向城镇转移，促进有能力在城镇稳定就业和生活的常住人口率先实现市民化。推进公共服务均等化，把进城农民纳入城镇住房保障体系，把农民工随迁子女义务教育纳入教育发展规划和财政保障范围，使进城农民进得来、住得下、生活得好。

五、抢抓机遇融入京津冀协同发展

从全面、全局、全过程认识和把握京津冀协同发展的重大战略意义，围绕“一枢纽四基地”功能定位，做好主动对接、积极承接、基础连接三篇文章。

(一)主动对接。按照即将发布的京津冀协同发展规划纲要，积极争取在交通、生态环保、产业、教育、医疗、商贸物流等国家专项规划以及全省协同发展规划和实施方案中，最大限度反映我市重大诉求，争取更多政策支持。积极推动体制机制创新，争列京津冀区域综合配套改革试点和国家中小城市综合改革试点。坚持引智引资并举，吸引京津高端人才向我市流动。在北京建立衡水创新孵化服务基地，主动对接北京高校和科研机构，打造协同发展的“桥头堡”。

(二)积极承接。继续实施“三个一百”工程。加快建设“蓝火计划”衡水基地，推动13个高校(院士)工作站尽快投用，加强高校企业双向交流对接合作。加大百家纺织服装企业进衡水和百家食品企业进衡水活动开展力度，今年外引落地项目都要确保达到40项以上。以把工业新区打造成为全国服装产业转移的聚焦点、京津纺织服装产业转移的首选地和河北纺织服装产业升级的主战场为目标，加大纺织服装企业外引力度，争取北京五木服装、北京方仕服装、天津纺织控股等项目早日落地开工。加快推进上好佳天津大湖整体搬迁等项目建设，争取北京红螺食品等项目签约落地，努力促进我市逐步向食品产业强市目标迈进。积极吸引京津教育、医疗、旅游休闲等产业向衡水转移，加快推进科技工程学校等院校与京津全面开展合作。鼓励与京津共建高水平的医疗机构和远程体检中心。密切关注京津产业转移目录，跑办对接央企、名企。推进铁塔产业与中国铁塔公司、工程橡胶产业与中国铁道总公司深度合作。

(三)基础连接。谋划与京、津、石等都市连通项目，使衡水纳入与京津冀区域核心都市的2小时、1小时交通圈，积极推动京衡客专、北京新机场南出口高速南延等项目前期工作，争取石衡高速、衡港高速阜城段、曲港高速衡水连接线等对接项目早日开工。加强区域合作，积极推动“石衡沧经济带”上升为省级战略，打造京津冀协同发展重要发展轴、新高地。六、坚持不懈改善生态环境

衡水湖是我市最宝贵的资源。把宝贵资源变为发展的核心优势，把改善环境作为最为重要的基础工作。积极开展环保攻坚年活动，认真落实新环境保护法，坚持源头严防、过程严管、后果严惩，标本兼治，提升环保工作整体水平，朝着蓝天净水绿树的目标不断迈进。

(一)全力打好大气污染防治攻坚战。着力在科学精准治霾上下功夫，加强重污染天气监测预警机制研究，持续打好压煤、治企、降尘、控车、禁烧五大战役，确保各项大气污染防治措施落实到位。加快改造淘汰10蒸吨及以下燃煤锅炉，加大散烧燃煤治理力度，支持每县建设一个洁净型煤配送中心和供应网络，确保到2017年洁净型煤覆盖面达到90%以上，全市煤炭消费量比2012年净削减100万吨。建立农村秸秆禁烧长效机制。全部淘汰黄标车，积极在公交车、出租车行业推广新能源汽车。整治农业面源污染，强化土壤污染防治。着力提升建筑能效，推动既有居住建筑供热计量和节能改造，全面推行绿色建筑行动，确保完成“建筑节能省”省定创建任务。进一步明确责任，确保不折不扣地完成全年节能减排任务。

(二)以壮士断腕的决心和勇气推进工业企业污染治理。严厉打击不法排污行为，关停违法排污、超标排污、恶意排污企业，对小电(热)镀、小浸塑、小化工等“土小”、“十五小”、“新六小”企业实施取缔关停。把环保治理作为企业技术改造的重要举措，推动传统产业转型升级。严格执行跨界断面水质目标考核和扣缴生态补偿金制度，提高生态补偿金扣缴比例，增加断面水质监测频次，确保包括污水处理厂在内的各类污水排放企业正常运行、达标排放，使水环境质量不断改善，滏阳河整体水质达到化学需氧量≤70毫克/升、氨氮≤10毫克/升，分别下降12.5%和50%。

(三)切实做好地下水超采综合治理和南水北调配套工程建设。完成2014年地下水超采综合治理项目，编制实施2015年地下水超采综合治理实施方案，确保压采地下水4.02亿立方米。抓好南水北调中线配套输水工程、地表水厂及配水管网建设，确保6月底前通水。通水后及时封填和限制使用受水区域城镇生活和工业地下水开采井。配合做好南水北调东线配套工程规划设计等前期工作。实行最严格的水资源管理制度，改革水价制度，加大节水型社会建设力度，增强全民节水意识，着力转变粗放的用水方式。

(四)加大衡水湖保护和造林绿化攻坚力度。启动实施利用德国政府贷款1200万欧元的衡水湖湿地保护与恢复项目。承办亚洲湿地保护网络研讨会。继续实施全国良好湖泊衡水湖生态环境保护项目。积极推进区域生态补偿机制改革试点工作。深化拓展“一人一亩林”成果，继续抓好环衡水湖、城市周边、主干道路、乡镇村庄等重点绿化工程，全年完成造林绿化10万亩。每县建设1至2个大体量、高水平的绿美廊道示范工程。完善多元投资营林造林机制，培育林业龙头企业，扶持发展巴迈隆等一批涉林企业。鼓励扩大核桃等经济林种植，在生态建设中推进林业产业化发展。

七、持续保障和改善民生

把促进社会公平正义、增进民生福祉作为政府工作的出发点和落脚点，解决好人民群众最关心最现实的利益问题。

(一)加强基本公共服务。统筹解决好高校毕业生、农村转移劳动力、退役军人、城市再就业人员等重点人群就业问题，千方百计保持就业稳定。整合城乡居民医疗保险资源，构建统一的城乡居民医疗保障制度。继续提高企业退休人员和城乡居民养老金、工伤保险待遇标准，新农合和城镇居民医疗补助标准提高到380元，实现新农合大病保险赔付出院即报。开展机关事业单位养老保险制度改革。做好60岁以上老年人数据库建设工作。建立80岁以上高龄补贴制度。加强农村五保供养服务机构建设，确保集中供养能力达到70%以上。全面建立临时救助制度，加大城市弱势群体帮扶力度。严格落实“双配建”政策，全年开工建设保障性住房1.99万套。解决41.95万农村人口饮水安全问题。

(二)大力发展社会事业。深入推进普通高中课程和综合素质评价改革，巩固基础教育领先优势。新建郑里马小学、庆丰街小学，改扩建中华街小学、和平路小学、第九中学，进一步缓解市区中小学班容量过大问题。改善贫困地区义务教育薄弱学校基本办学条件，促进教育公平。桃城区、冀州市、枣强县、工业新区和滨湖新区要力争通过义务教育基本均衡县省级评估和国家认定。鼓励社会力量兴办职业教育，加快建设职业教育强市。支持衡水学院向应用技术大学转型，衡水职业技术学院、市直中等专业学校和各县市区职教中心办好经济社会需要的特色优势专业。优化卫生资源配置，完善区域卫生规划，健全农村三级医疗卫生服务网络。加快市博物馆、图书馆搬迁，大力提升县级图书馆、文化馆、乡镇综合文化站和农村文化大院建设水平，积极构建现代公共文化服务体系。办好2015衡水湖国际马拉松赛，举办市第三届运动会，打造城市社区15分钟健身休闲圈，推进市全民健身中心和场地自行车训练馆建设，支持市体育学校建设，以“篮球城”、“长跑城”、“健步城”为重点，加快打造体育名城。加大扶贫攻坚力度，完成84个贫困村的特色产业增收项目，确保10万贫困人口稳定脱贫，枣强、故城两个省定贫困县力争脱贫出列。

(三)积极创新社会治理。做好信访工作，引导群众依法有序表达诉求，及时就地解决群众合理诉求，严厉惩治拖欠农民工工资行为。落实安全生产“党政同责、一岗双责”，扎实开展预防较大以上生产安全事故专项行动，深化“1+1”安全生产专项整治，加大重点行业、重点领域安全生产整治力度，推动安全生产打非治违常态化。强化公共安全，抓好道路交通、消防和人员密集场所安全保障，坚决遏制重特大事故发生。加强食品药品安全监管。依法打击各类违法犯罪活动，为人民群众创造和谐稳定、安全有序的生产生活环境。

继续支持国防和军队建设，加大国防后备力量和国防动员建设力度，深入开展国防教育，做好双拥共建工作，推进军民融合深度发展。支持工会、共青团、妇联等人民团体发挥桥梁纽带作用，做好新闻出版广电、老龄、妇女儿童、残疾人、气象地震防灾减灾、民族宗教、人民防空、邮政通信、统计、方志档案、外事侨务和涉台事务等工作。高质量编制“十三五”规划。

八、全面加强法治政府建设

积极适应全面推进依法治国新要求，坚决维护宪法法律权威，大力推进依法行政，加快建设职能科学、权责法定、执法严明、公开公正、廉洁高效、守法诚信的法治政府，把法治思维和法治方式贯穿政府工作全过程。

依法履行政府职能。坚持法定职责必须为、法无授权不可为，把政府职能切实转向经济调节、公共服务、市场监管、社会管理和环境保护。加快落实清单制度，在已公布“负面清单”和“权力清单”基础上，6月底前完成“监管清单”和“责任清单”编制并向社会公布。完成市县政府机构改革。深化行政审批制度改革，开展建立行政审批局试点工作，完善“三级平台”、“两个代办”，推进网上服务，实现行政审批事项网上一体化办理和联合审批。对法定职责范围内的行政事务，要勇于负责、敢于担当，坚决纠正不作为、乱作为，坚决克服懒政、怠政，坚决惩处失职、渎职。

健全依法决策机制。出台实施行政决策程序规定，建立健全公众参与、专家论证、风险评估、合法性审查、集体讨论决定重大事项决策程序，完善政府法律顾问制度，实施重大行政行为备案办法，建立重大决策终身责任追究制度和责任倒查机制。完善重大事项向人大报告制度，积极采纳人大、政协调研建议，吸取各民主党派、工商联、无党派人士意见，充分发挥离退休老同志、专家学者和各类智库作用，加强政府协商，增强决策透明度和公众参与度。加强对行政决策落实的监督，确保决策执行落实到位。

严格规范行政执法。积极推进综合执法，合理配置执法力量，减少行政执法层级，整合执法队伍种类，努力解决市、区、部门之间职能交叉问题。深入推进城市管理领域相对集中行政处罚权，理顺城管执法体制。加大食品药品、安全生产、环境保护、征地拆迁、民间融资等重点领域执法力度，维护群众合法权益。大力推进“六五”普法工作。完善执法程序，细化、量化市本级行政裁量标准，提高执法效率和规范化水平。全面落实行政执法责任制，严格实行执法责任追究制度。

强化权力制约监督。自觉接受市人大的法律监督、工作监督和市政协的民主监督，诚恳接受社会监督和舆论监督。加强对政府内部权力的制约，对权力集中的部门和岗位，实行分事行权、分岗设权、分级授权，定期轮岗，强化内部流程控制，防止权力滥用。加强行政复议和行政应诉工作，强化层级监督，保障和支持审计、监察等部门依法独立行使监督权。完善纠错问责机制，健全问责方式和程序，严肃追究违纪违法行政工作人员的责任。深入推进政务公开，让权力在阳光下运行。

各位代表，面对新常态和新要求，我们将始终践行群众路线，把对人民负责作为第一准则，把让人民满意作为首要目标，努力建设负责任、敢担当、办实事、有作为的政府。

我们将以更大的力度推进改革创新。坚持把改革作为适应经济发展新常态的根本举措，依靠改革破解制约发展的体制机制障碍，推进治理体系和治理能力现代化；依靠改革妥善处理政府与市场、社会的关系，把适宜放给市场的权限放给市场，把适宜放给社会的权限放给社会，把适宜放给基层政府的权限坚决下放。把解放思想、锐意创新贯穿政府工作全过程，在更大范围、更宽领域、更高层次上推动工作创新。

我们将以更实的作风服务群众。巩固深化党的群众路线教育实践活动成果，持之以恒推进作风建设，着力解决“四风”突出问题，切实为基层、企业和群众解决实际问题。坚持从严治政，狠抓工作落实，持续开展对全市稳增长、调结构、促改革、惠民生、防风险政策措施落实情况的督促检查和跟踪审计，促进政令畅通，切实提高政府执行力。

我们将以更大的决心反腐倡廉。严格执行“八项规定”、“约法三章”和新预算法，牢固树立过“紧日子”的思想，从严控制“三公”经费支出。完成机关公务用车制度改革。认真履行党风廉政建设主体责任，以锲而不舍、驰而不息的决心，不断提高拒腐防变和抵御风险能力。强化重点领域和关键岗位监管，严肃查处违纪违法案件，树立为民、务实、清廉的政府形象。

各位代表!面对全市人民的信任和重托，面对新的挑战和考验，我们倍感使命在肩、责任重大。让我们紧密团结在以习近平同志为总书记的党中央周围，在省委、省政府和中共衡水市委的正确领导下，锐意进取，攻坚克难，为实现跨越赶超、绿色崛起、全面建成小康社会目标而努力奋斗!
